# Structural and functional specificity of H3K36 methylation

**DOI:** 10.1186/s13072-022-00446-7

**Published:** 2022-05-18

**Authors:** Ulysses Tsz Fung Lam, Bryan Kok Yan Tan, John Jia Xin Poh, Ee Sin Chen

**Affiliations:** 1 Department of Biochemistry, Yong Loo Lin School of Medicine, National University of Singapore, Singapore, Singapore; 2 National University Health System (NUHS), Singapore, Singapore; 3 NUS Center for Cancer Research, Yong Loo Lin School of Medicine, National University of Singapore, Singapore, Singapore; 4 Integrative Sciences & Engineering Programme, National University of Singapore, Singapore, Singapore

**Keywords:** Set2, SETD2, NSD2, NSD3, ASH1L, H3K36, Methylation

## Abstract

The methylation of histone H3 at lysine 36 (H3K36me) is essential for maintaining genomic stability. Indeed, this methylation mark is essential for proper transcription, recombination, and DNA damage response. Loss- and gain-of-function mutations in H3K36 methyltransferases are closely linked to human developmental disorders and various cancers. Structural analyses suggest that nucleosomal components such as the linker DNA and a hydrophobic patch constituted by histone H2A and H3 are likely determinants of H3K36 methylation in addition to the histone H3 tail, which encompasses H3K36 and the catalytic SET domain. Interaction of H3K36 methyltransferases with the nucleosome collaborates with regulation of their auto-inhibitory changes fine-tunes the precision of H3K36me in mediating dimethylation by NSD2 and NSD3 as well as trimethylation by Set2/SETD2. The identification of specific structural features and various *cis*-acting factors that bind to different forms of H3K36me, particularly the di-(H3K36me2) and tri-(H3K36me3) methylated forms of H3K36, have highlighted the intricacy of H3K36me functional significance. Here, we consolidate these findings and offer structural insight to the regulation of H3K36me2 to H3K36me3 conversion. We also discuss the mechanisms that underlie the cooperation between H3K36me and other chromatin modifications (in particular, H3K27me3, H3 acetylation, DNA methylation and N^6^-methyladenosine in RNAs) in the physiological regulation of the epigenomic functions of chromatin.

## Introduction

Eukaryotic genomic DNA is packaged into structural units called nucleosomes. Each nucleosome consists of 146 base pairs (bp) of DNA that wrap around (1.6 turns) an octameric complex comprising two of each of histones H2A, H2B, H3 and H4 [1, 2]. In most eukaryotes, there exists a linker histone H1 to stabilize the chromatin structure and allow for further compaction of chromatin into more complex higher-order structures [2–5]. The amino acid residues of the core histone proteins are heavily decorated with post-translational modifications (PTMs), particularly on the unstructured histone tail domains, and these modifications help to regulate chromatin compaction and govern the availability of docking sites for chromatin-modifying factors [1, 6]. PTMs such as methylation, phosphorylation, acetylation, SUMOylation and ubiquitination occur via the transfer of chemical moieties onto specific residues of histone proteins [7] that engage in crosstalk and influence various DNA metabolic processes, including gene transcription [7–10], DNA damage repair [11, 12], specialized chromosomal loci assembly [13, 14], and cell cycle progression [15–18].

**Fig. 1:**
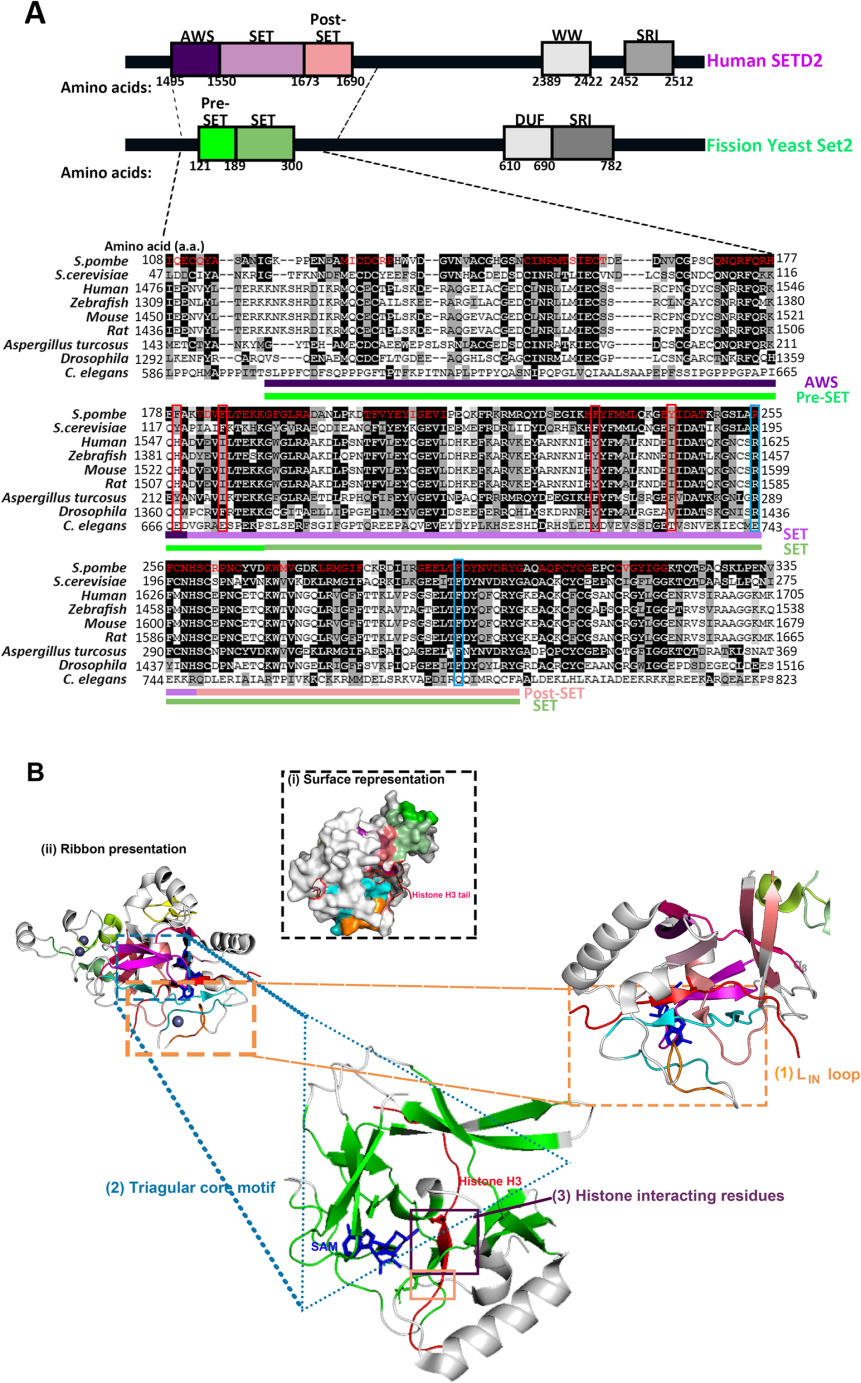
Set2/SETD2 is generally conserved among fungi and metazoans. **A** Multiple sequence alignment (MSA) of Set2 homologs across fungi and metazoan species. MEGAX software and NCBI MSA viewer 1.13.1 [230] were used to generate an alignment of the primary amino acid sequences of Set2/SETD2 homologs from fission yeast, budding yeast, fungi *Aspergillus turcosus*, and metazoan species including human, rat, mouse, zebrafish, fruitfly, and nematode worm *C. elegans*. 35% of the human SETD2 amino acid sequence is conserved in fission yeast Set2 , even for sequences beyond the catalytic SET domain. 53% of the amino sequence in the catalytic SET and post-SET domains is identical in fission yeast Set2 and human SETD2. Residues that are generally conserved across species are indicated in red. Residues that are identical or similar in polarity across species are, respectively, highlighted in black or grey. Conserved “decision-making” residues that regulate the degree of methylation are circled in blue. Non-conserved aromatic residues that make contact with histone tails and possibly participate in methylation regulation are circled in red. Domain structure of fission yeast Set2 and human SETD2 are also shown to indicate the relative amino acid positions of the MSA sequences in the SETD2 homologues. **B** Conserved motifs in human SETD2 SET-domain. **i** Surface representation: sections of conserved motifs are highlighted (green, teal, cyan, and orange). Pymol visualization is derived from the crystallized structure database in the Protein Data Bank, entry 5V21 [74]. **ii** Projection of the conserved sequence of yeast Set2 and human SETD2. The SET domain crystalized structure highlights (1) a regulatory L_IN_ loop, (2) a triangular core motif separated from the catalytic site, and (3) histone-interacting residues (refer Figs. 3 and 4). Conserved residues in the triple β-sheet in the triangular core of the SETD2 SET domain (green) endow the SET domain with its recognizable triangular shape, which maintains the structure of the domain. Conserved L_IN_-loop (teal, cyan and orange) and α_8_ (short, white α-helix region) in the closed conformation secure the histone tail in position for methylation.

Lysine 36 of histone H3 (H3K36) can be modified by mono-, di- or trimethylation (H3K36me1, H3K36me2 and H3K36me3, respectively). In human, this is orchestrated by a redundant series of enzymes: nuclear receptor-binding SET domain (NSD) protein 3, which only catalyzes monomethylation in vivo; NSD1, NSD2,ASH1L and MYND domain-containing 2 (SMYD2), which catalyze dimethylation in vivo; and SET domain-containing 2 (SETD2) , which catalyzes trimethylation in vivo; albeit, SETD2 is capable of catalyzing all forms of methylation in vitro [19–32]. Yeasts, such as budding yeast *Saccharomyces cerevisiae* and fission yeast *Schizosaccharomyces pombe*, encode a single H3K36 methyltransferase (HMTase) in their genomes, known as Set2, which bears a high sequence similarity to SETD2 (Fig. 1A) and is responsible for generating all three forms of histone methylation in yeast [12, 33–35]. Like mammalian H3K36 methyltransferases, yeast Set2 is recruited to chromatin during transcription elongation to catalyze H3K36me in a co-transcriptional manner [33, 36–38].

Proper H3K36me epigenome function is critical for maintaining genomic stability, with defects in this process widely observed to be associated with human diseases, including prenatal developmental disorder [39, 40], and cancer [41–45]. These conditions arise presumably due to defects in processes linked with transcription, splicing [46–49], and cell cycle regulation [15–18]. In yeast cells, mutations in Set2 or H3K36 lead to a decreased lifespan, presumably by reasons of a global loss of histone methylation [44] and an inability to mount a proper response to environmental stressors like genotoxic insult [12, 16] or nutrient deprivation [50].

Here, we consolidate the structural, functional and physiological research—garnered predominantly from studies in yeast Set2 and human NSD2, NSD3, ASH1L and SETD2—pertaining to the catalysis of H3K36 methylation, and cross-reference yeast studies against those carried out using human SETD2 and other H3K36 methyltransferases.

## Physiological roles of Set2/SETD2 in DNA damage repair and tumor suppression

SETD2 is a known tumor suppressor and is involved in several molecular pathways that maintain genomic stability. SETD2 mutations are associated with progression, recurrence, and survival rates, especially among patients with clear cell renal cell carcinoma (ccRCC) [45, 51–53]. We reviewed this topic in detail recently, so here we will only briefly discuss some of these findings [54]. Mutations affecting H3K36 methylation—particularly SETD2 truncation mutations—account for > 30% of pediatric high-grade gliomas [54, 55]. SETD2 loss-of-function mutations were observed in 10% of primary and 30% of metastatic ccRCC tumors, whereas H3K36 methylation is significantly reduced in ccRCC cell lines and patient samples [56]. In ccRCC, an R1625C point mutation in SETD2 destabilizes SETD2 protein, reduces its capacity for substrate binding, diminishes H3K36me3, and delays the DNA damage response, with evidence of reduced γH2A.X foci formation in an H3K36me3-dependent manner. Interestingly, introduction of the equivalent mutation in budding yeast (R195C) results in a similar attenuation of H3K36me3; albeit it affects less H3K36me1 and H3K36me2 levels, implicating a preferential role of R1625 in the formation of H3K36me3 [57]. In contrast, R2510H mutation within the SETD2 SRI domain—another common ccRCC-associated mutation—has no effect on H3K36me3. This may indicate that the interaction between SETD2 and RNA polymerase II (RNAPII) is a functionally discrete mechanism in ccRCC carcinogenesis [57]. In the case of acute lymphoblastic leukemia, H3K36me3 is enriched on most genes regulated by leukemia-associated transcription regulator MLL. Indeed, the downregulation of H3K36me3 by SETD2 mutation can attenuate leukemia cell proliferation [58].

At the molecular level, Set2/SETD2 regulates several processes that are essential for the maintenance of genomic stability, including response and repair of various types of DNA damage, alternative splicing and ensuring proper progress of the cell cycle. These three processes may compositely underlie major tumor suppression pathways regulated by SETD2 in human cancers.

H3K36 methylation has been linked to homologous recombination (HR) in response to various types of DNA lesions. For example, in the case of UV-induced DNA damage, SETD2 is involved in the recruitment of 53BP1 to damaged DNA loci via its interaction with γH2AX and H3K36me3 [59]. In contrast, at strand breaks, lens epithelium-derived growth factor (LEDGF; p75) is recruited onto DNA double-stranded breaks (DSB) via its interaction with H3K36me3 marks to generate single-stranded break overhangs with the C-terminal binding protein interacting protein (CtIP) nuclease [60, 61]; RAD51 and PALB2 are then recruited via H3K36me3 binding to stabilize and guide strand invasion for DNA repair [59, 62–65]. In fission yeast, Set2 has been reported to mediate the timely localization of the HR factor Rhp54 as well as nucleotide excision repair factor Rhp23 when the cells are exposed to DNA alkylating damage following methyl methanesulfonate (MMS) treatment [12]. These DNA damage response factors have been shown to be targeted to gene loci such as *brc1*^+^—which encodes a BRCT domain factor, similar to human BRCA1—to elicit the repair of damaged DNA [12].

Human SETD2 physically interacts with splicing factors, U2AF and SF-1, as well as heterogeneous nuclear ribonucleoproteins via SETD2-hnRNP Interaction (SHI) domain to regulate splicing [66]. Thus, not surprisingly, deregulation of alternative splicing has been shown to enhance tumorigenesis [47, 67–69]. Knockout of *SETD2* in intestinal cells of a colorectal cancer mouse model deregulated the alternative splicing of Wnt/β-catenin signaling genes (e.g., disheveled segment polarity protein 2) to promote metastasis [47]. Primary human kidney tumors bearing SETD2 mutations and hosting a global reduction in H3K36me3 were found to have disruptions in processes concerning mRNA processing, intron retention, and splicing in ~ 25% of genes in the genome [70]. Furthermore, the knockdown of SETD2 in human gastric cancer cell lines was shown to result in the accumulation of aberrantly spliced transcripts of the mismatch repair gene hMLH1, suggesting that interfering with the alternative splicing of DNA damage repair genes may also underlie defective DNA damage responses in the disrupted function of SETD2 [67].

Fission yeast *set2* null mutant showed reduced phosphorylation of the DNA damage checkpoint kinase Chk1 (similar to mammalian CHK2), suggesting that Set2 also regulates proper activation of the DNA damage checkpoint [12]. A similar effect on DNA damage checkpoint was also observed in the mammalian system, in which a leukemia-associated SETD2 F2478L point mutation caused a decrease in the activating phosphorylation of checkpoint kinases CHK1 and CHK2 and cyclin-dependent kinase (CDK)-inhibiting WEE1 kinase [18]. Transcriptomic analysis in the SETD2 F2478L leukemic cells revealed a downregulation in the genes associated with G2/M progression, DNA replication and p53 apoptotic pathways, suggesting that SETD2 may control cell cycle-related transcription programs. Indeed, both human SETD2 and fission yeast Set2 have been reported to regulate the expression of ribonucleotide reductase (RNR) complex genes to ensure the precise progression of cells through S-phase [71, 72]. In osteosarcoma cells treated with WEE1 kinase inhibitor AZD1775, SETD2 knockout induced an accumulation of cells in S-phase because of reduced RRM2 RNR subunit expression; this, in turn, induced collapse of the DNA replication fork [71]. SETD2 also regulates the cell cycle via phosphorylation of Lys-40 of α-tubulin to mediate proper mitotic spindle formation and cytokinesis [15].

## Domain structure in SETD2 homologs

 Set2 and SETD2  are modular enzymes bearing several defined molecular motifs that coordinate the molecular functions of the H3K36 methyltransferases (Fig. 1A and B). The primary amino acid (a.a.) sequence of mammalian SETD2 is highly similar to the sequences of fly and yeast Set2 proteins (Fig. 1A); albeit, the lengths of these proteins vary greatly from 733 a.a. in *Saccharomyces cerevisiae* Set2 protein to 2,537 a.a. in *Mus musculus* SETD2 [32, 57]. Despite the disparity in length, the function of these factors is highly conserved, owing to the dominant function of the SET domain. Indeed, the H3K36 methylation profiles on the genome are remarkably similar across Set2/SETD2 homologs, even across different orders of life [32, 73].

Set2/SETD2 homologs identified by PSI-BLAST search show similar domain architecture, consisting of post-SET, SET, Associated With SET (AWS), and Set2-Rpb1 interaction (SRI) domains [33, 57, 74] (Fig. 1A). The AWS [75], SET, and post-SET domains are necessary for the catalytic function of Set2/SETD2 in methylating H3K36 [12, 76]. In contrast, the C-terminal SRI domain drives the binding between Set2/SETD2 and the C-terminus of the Rpb1 subunit of RNAPII. The SRI motif comprises three α-helices that recognize and bind to the phosphorylated Ser-2 residue within the hepta-repeats of the hyperphosphorylated CTD of Rpb1 [36, 37, 77]. In vitro electromobility assays have been used to confirm that the SRI domain of budding yeast Set2 binds to nucleosomal linker DNA and hence determines Set2 enzyme substrate specificity [78]. Deleting the SRI domain has no effect on Set2 chromatin localization, indicating that there are other mechanisms regulating chromatin binding of Set2/SETD2 [79]. In fact, the SET domain and flanking regions are reported to bind single-stranded nucleic acids, including RNA and single-stranded DNA [80], whereas the N-terminus of Set2 interacts with histone H4 [80], thus contributing to the stability of the protein and enhancing its binding to chromatin.

In H3K36 HMTases of mammals, nematode worm and fly, the tryptophan-tryptophan (WW) and the coiled-coil (CC) domains lie distal to the post-SET domain and function in protein–protein interactions. The WW domain binds a poly-proline (Pro) stretch within itself to constitute an intramolecular auto-inhibitory module, which competes with the binding between the WW domain of SETD2 and the Pro-rich region of the Huntingtin protein [81]. The CC domain, on the other hand, promotes dimerization within another auto-inhibitory motif to regulate the extent of methylation [32, 78, 82].

Budding yeast Set2 also contains an auto-inhibitory domain (AID), which lies between the catalytic SET domain and the RNAPII-binding SRI domain. Truncation of the AID allows mutated Set2 to bind to unphosphorylated RNAPII, thereby generally promoting Set2 catalytic activity and enhancing cryptic transcription [78]. However, this domain is not conserved in fission yeast or human. In addition, the detailed regulatory mechanism of the AID remains mostly unknown [32].

## Histone H3K36 di-methyl transferases

### Domain structure of NSDs and ASH1L

**Fig. 2:**
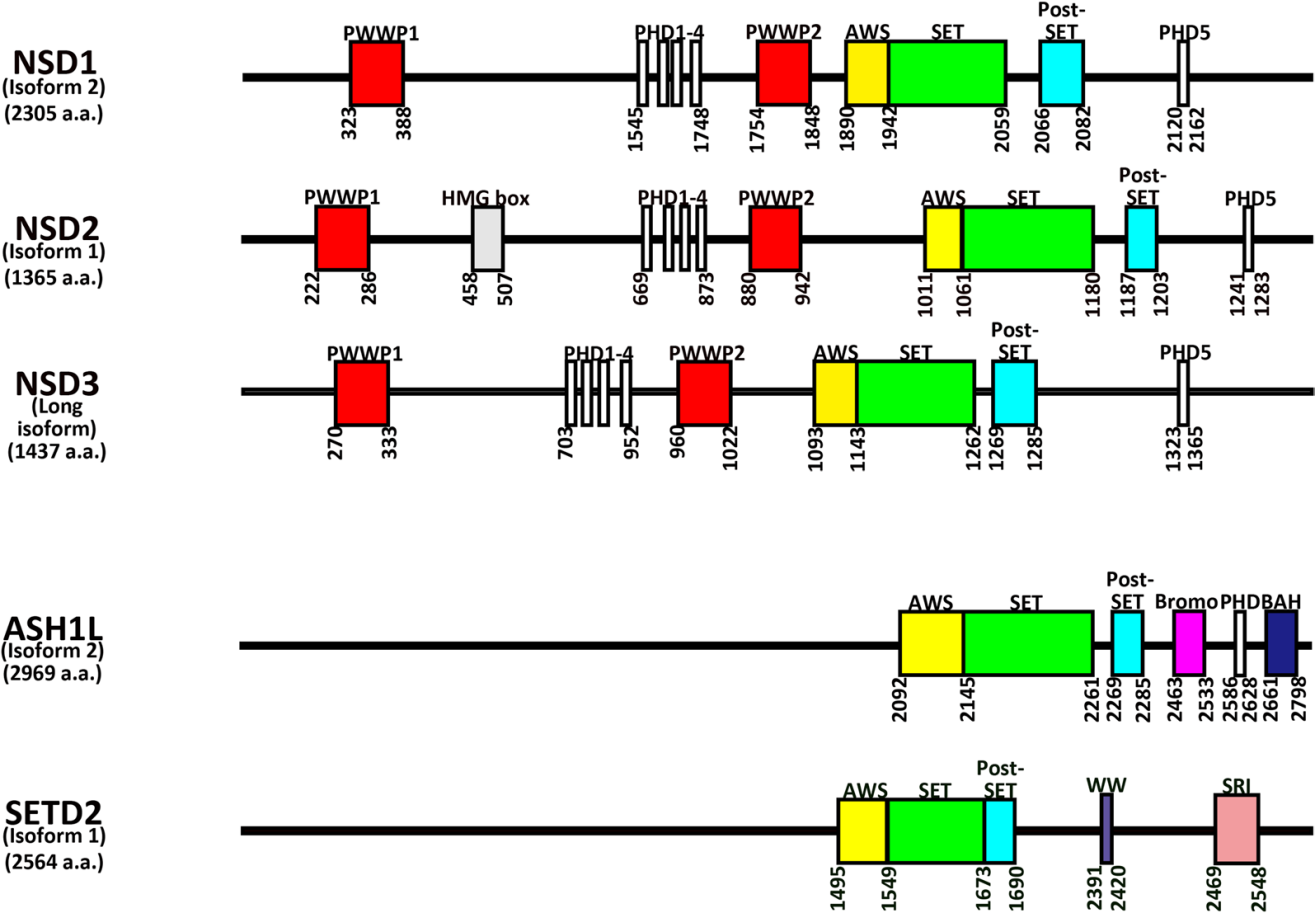
Schematic diagram detailing the domain structure of common isoforms of NSD methyltransferases, ASH1L and SETD2. Numbers listed vertically refer to the relative amino acid positions of the domains in the different methyltransferases

Histone H3K36 can be dimethylated by several enzymes [27] including ASH1L, NSD2 and NSD3; these NSD proteins have been structurally studied in detail. The NSD family of proteins comprise the AWS, SET and post-SET domains, which compositely form the catalytic regulatory domain that is shared with Set2 and SETD2 trimethylase (Fig. 2). In addition, the family also contains three types of chromatin-associating domains, namely the plant homeodomain (PHD) domains: four PHD motifs are flanked by two proline-tryptophan-tryptophan-proline (PWWP) domains at the N-terminus (PWWP1 and PWWP2), with the fifth PHD motif located in the C-terminus, distal to the AWS-SET-post-SET catalytic domains. A high mobility group (HMG) motif is found solely in NSD2 (Fig. 2). The PWWP, PHD and HMG domains associate with various components of chromatin: PWWP1 in NSD2 can bind with methylated H3K36 to stabilize chromatin association of the enzymes and enable propagation of H3K36me3 [83]. PHD motifs are critical in sustaining the catalytic activity of NSD2; indeed, one study showed that ablation of the second PHD delocalized NSD2 from the nucleus to the cytoplasm, and this mislocalization correlated with complete abolishment of enzymatic activity [84]. The HMG box confers the nuclear localization of NSD2 by mediating its interaction with the DNA-binding domain of androgen receptor (AR) [85]. Overexpression of NSD2 provokes AR transcription, which in turn induces *RAS* signaling and promotes prostate cancer progression [86, 87].

ASH1L, of the Trithorax group of proteins, is another H3K36 di-methylase in fly and mammalian cells. ASH1L also shares the AWS-SET-post-SET catalytic domains, but differs to the NSD proteins, as it hosts a bromodomain and a BAH domain in its C-terminus (Fig. 2) [88, 89].

#### Roles of H3K36 dimethylases in oncogenesis and cancer progress

Overexpression of the NSD family proteins is shown to be oncogenic. NSD1 promotes the progression of acute myeloid leukemia, multiple myeloma, and lung cancer, as well as cancer cell migration and invasion in hepatocellular carcinoma (HCC), pediatric glioma, and breast cancer [90–95]. The carcinogenic potential of NSD1 is mediated via activation of Wnt/β-catenin signaling [90, 94].

NSD2 overexpression is linked with tumor aggressiveness [96] in cancers of the breast [97], cervix [98], lung [99], kidney [100], head and neck [101], brain [91], blood [102], colorectum [103], prostate, skin [96], and ovary [24]. Carcinogenesis associated with changes in the expression of NSD2 is also linked with cell cycle dysregulation [102], VEGF-A-mediated angiogenesis [104], hematopoietic stem cell differentiation [105], metastasis [91], chemoresistance (in osteosarcoma) [106], and the expression of various oncogenes (e.g., *SYK*, *PTPN13* and *ETV5* in multiple myeloma) [107]. Gain-of-function mutations (E1099K and T1150A) in the SET domain of NSD2 have been associated with the enhanced enzymatic activity of NSD2 in mantle cell lymphoma and pediatric acute lymphoblastic leukemia, in which they cause destabilization of the auto-inhibitory loop responsible for keeping signaling in check (refer below) [108, 109]. E1099K mutation is also found in 70% of patients with leukemia who experience relapse, and has been linked with aberrant global DNA methylation profiles in these patients [108, 110].

NSD3 is overexpressed in 58% of patients with advanced squamous cell carcinoma of the head and neck [92, 101]. NSD3 is also connected with metastatic cancers of the breast [111, 112], colorectum [113], pancreas [114] lung [115], and bone [116]. A short non-catalytic isoform of NSD3 that retains only the PWWP domain can promote oncogenesis by antagonizing proteasome-mediated degradation of the MYC oncogene [117]. Like NSD2, mutations in the SET domain of NSD3 (E1181K and T1232A), which counteract the auto-inhibition mechanism of H3K36 methylation, are associated with mantle cell lymphoma and chronic lymphocytic leukemia [118, 119].

ASH1L is involved in a global genomic nucleotide excision repair regulation and is recruited by DNA damage specific DNA-binding protein 2 (DDB2) to promote cyclobutane pyrimidine dimer excision [120]. There is evidence to show that, in acute leukemic conditions, translocation of *MLL1* leads to deletion of *ASH1L*, followed by the transcriptional activation of various oncogenes (e.g., *HOXA9*) and cancer transformation [89, 121, 122]. ASH1L overexpression is associated with the progression and development of breast, liver, and thyroid cancers [123–125].

#### Neurodevelopmental roles of H3K36 dimethylases

*NSD* genes are important for pre- and postnatal neurodevelopment and their loss-of-function mutations relate to neurological syndromes, particularly Soto’s and Wolf–Hirschhorn’s syndromes [126, 127]. Soto’s syndrome, which occurs in 1/14,000 births and is associated with intellectual disability, facial deformation, and overgrowth phenotypes, is genetically mapped to *NSD1* haploinsufficiency [126, 128]. Cells derived from a patient with Soto’s syndrome hosting an NSD1-inactivating mutation was shown to have a global redistribution of the DNA methyltransferase DNMT3A, along with promoter DNA hypomethylation, dysregulated synapse formation, and dysregulated neurodevelopmental gene expression [129–131]. Moreover, individuals hosting *NSD1* whole-gene deletions exhibited early-onset cerebrovascular diseases [132]. This phenotype was recapitulated by deletion of an *NSD1*-like gene in fly, with developmental symptoms accompanied by global H3K36me2 reduction, defective motor and memory functions, and body size overgrowth [133]. Conversely, overexpression of the NSD1 homolog induced neuronal apoptosis and larval locomotive defects [134].

Mutation or deletion of NSD2 is believed to underlie Wolf–Hirschhorn’s syndrome, which is clinically characterized by pre- and postnatal neurodevelopmental disability and hypotonia [127, 135]. NSD2 point mutations at C869Y (in PHD domain), P895L (in PWWP domain), K1019R (in AWS domain), E1091K, E1099K, and S1137F (in SET domain) are associated with a global decrease in H3K36me2 levels in patients with Wolf–Hirschhorn syndrome. In particular, C869Y mutation in the PHD domain disrupts the interaction between NSD2 and its zinc cofactor, which, in turn, disrupts proper protein folding and its subsequent catalytic function. In contrast, P895L mutation in the PWWP domain causes steric clashes within the molecule, whereas K1019R mutation in the AWS domain inhibits the formation of a mobile loop in the catalytic domain that is necessary for methylation function [136].

ASH1L has a role in synaptic plasticity via H3K36me at the promoter of neurexin-1α in response to action potentials [137]. Mutations in ASH1L are clinically associated with intellectual disability [138] as well as early-onset Lewy body dementia [139]. In addition, A2780P point mutation in the BAH domain of ASH1L is associated with autism spectrum disorder [140–143], whereas Y2077F (N-terminal region) and S2200G (SET domain) point mutations are bioinformatically linked to Tourette’s syndrome (TS) [144].

## Determinants of H3K36 methylation catalysis

Recent cryo-EM studies on the interactions between H3K36M and Set2, NSD2 or NSD3 have uncovered universal structural determinants of H3K36me catalysis, refining previous knowledge from X-ray diffraction and cryo-EM studies of SETD2 [75, 109, 119, 145]. Indeed, several structural features have been identified as being critical for regulating H3K36me reactions, in addition to positioning of the histone H3 tail within the catalytic pocket of the enzyme and the auto-inhibitory mechanism of human SETD2 and filamentous yeast *Chaetomium thermophilum* Set2 [75, 145]. Of note, the auto-inhibitory loop has been identified within the dimethylases NSD2 and NSD3 [119, 146]. Furthermore, a hydrophobic surface constructed by the C-termini of histone H2A, the αN helix of histone H3, and the linker DNA of the nucleosome plays an important role in regulating the catalytic activity of Set2/SETD2. Here, we explore each of these determinants in more detail:

### Structural motifs in Set2/SETD2 in H3K36me catalysis

**Fig. 3:**
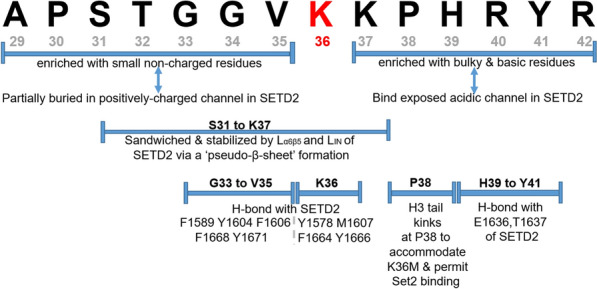
SETD2 interaction with K36 and its flanking residues of Histone H3.3 residue from A29-R42. Note that the summary is based on results derived from histone H3 peptides with M36K substitution, which stabilizes protein interaction [75, 145]

**Fig. 4:**
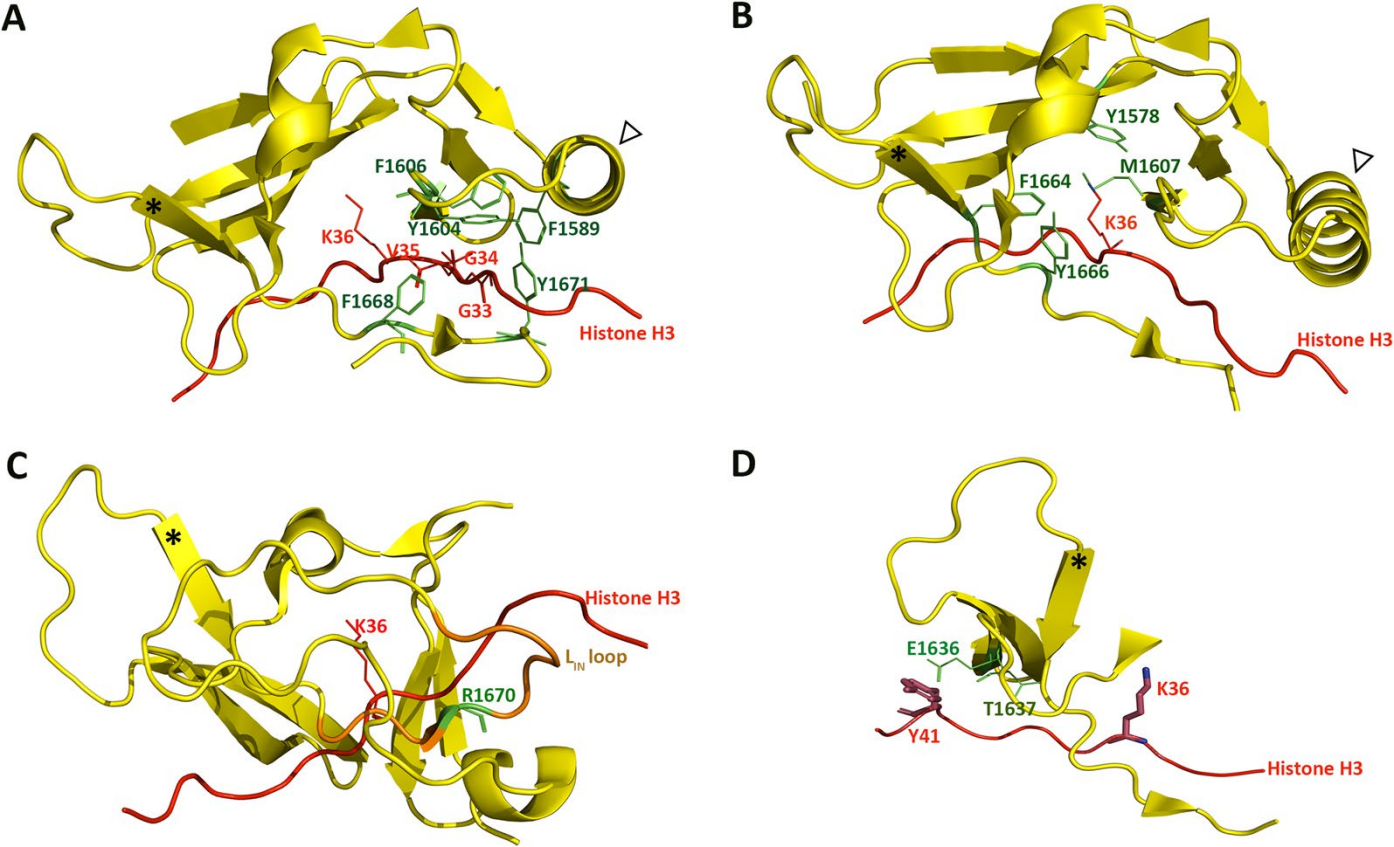
Conserved histone-interacting residues in the SET domain of SETD2. Asterisk (*) and white arrow in the figure, respectively, label the position of SETD2 β15 beta sheet and α6 helix to depict relative orientation of SETD2 crystalized structure in different sub-figures. **A** F1589, Y1604, F1606, F1668, and Y1671 (green) interact with G33-V35 (dark red) of the histone H3 peptide (red) via hydrogen bonding. **B** Key catalytic residues Tyr-1578, Met-1607, Phe-1664 and Tyr-1666 (green) of the SETD2 SET domain (yellow) surround the K36/M36 residues (dark red) of the histone H3 peptide (red). **C** Key auto-inhibitory residue R1670 (green) of the L_IN_ loop (brown) in SETD2 SET domain (yellow) is in proximity to K36 (dark red) on histone H3 peptide (red). **D** E1636 and T1637 (green) interact with Y41 (dark red) of the histone H3 peptide (red) via hydrogen bonding. Pymol visualization is derived from the crystalized structure database in the Protein Data Bank entry 5V22 [160].

Set2/SETD2 is a modular enzyme that hosts several well-conserved domains. There is a 35% similarity in the a.a. sequences of the human SETD2 and both budding and fission yeast Set2, with nearly half of these conserved residues found within the catalytic pre-SET and SET domains (Fig. 1A). Projection of the conserved sequences of the SET domain of yeast Set2 onto human SETD2 SET domain (Fig. 1B) highlights three features: (I) a regulatory L_IN_ loop, (II) a triangular core motif juxtaposing the catalytic site, and the presence of histone-interacting residues (Figs. 1B, 3, 4A and B) [145]. These features pose major impact on catalysis and the regulation of histone methylation [145, 147, 148].

- (I) The regulatory LIN loop

Various conserved residues are found within key regions of the L_IN_-loop. This loop connects the SET domain with the post-SET motif and is involved in loading and positioning the H3 histone tail (Fig. 1B) [145]. Arg-1670 (SETD2 numbering) appears to serve as a key auto-inhibitory residue that dictates the opened and closed conformations of the H3K36 access pocket [145]. This residue aligns with budding yeast Arg-240 and fission yeast Arg-300, and is in close proximity to Lys-36 of the histone H3 tail (Figs. 1B, 4C) as well as other key catalytic residues critical for HMTase activity: Met-1607, Phe-1664, and Tyr-1666 in human SETD2 (Met-177, Phe-234 and Phe-236 in budding yeast; Met-237, Phe-294 and Tyr-296 in fission yeast Set2) [74, 145] (Figs. 1B, 4B).

Yang et al. (2016) observed that the L_IN_ loop can adopt three major states—closed, partially open and open—in different stages of the auto-inhibitory model [145]. The ‘closed’ L_IN_ loop assumes a ‘compacted conformation with Arg-1670 inserted into the space within the catalytic center that is usually occupied by Lys-36 when histone H3 is loaded into SETD2. In this way, the L_IN_ loop acts to inhibit the enzymatic activity of SETD2 by occluding the H3K36 substrate-binding site (Fig. 4C). In the ‘partially open’ state, Arg-1670 is displaced outward to prime the catalytic pocket for entry of the histone H3 tail. The L_IN_ loop in the ‘open’ state adopts an extended conformation with Arg-1670 shifted approximately 4.5A away to create sufficient space for loading of the histone H3 N-terminus into the substrate channel of the SETD2 catalytic site (Fig. 4C). This binding of the H3-tail peptide subsequently triggers structural organization of the residues at L_IN_ loop and the post-SET domain into a ‘knot-like’ structure that stabilizes the docked H3-peptide [145].

Similar auto-inhibitory conformational changes were also found in the NSD2, NSD3 and ASH1L dimethylases [27, 109, 119, 149]. The compacted auto-inhibitory loop of NSD2 and NSD3 was observed to ‘open up’ upon binding of the enzymes to the nucleosome, thus making room for the K36M peptide to dock into the catalytic groove [109, 119]. Furthermore, the transition between the ‘open’ and ‘closed’ states is actively regulated via the interaction of transacting factors. Specifically, the chromatin remodeler *MORF4*-related gene on chromosome 15 (MRG15) binds to a FQLP motif proximal to the AWS-SET domain of ASH1L and, in so doing, displaces the auto-inhibitory loop [149]. In fly, loss-of-function mutations in *MRG15* and *ASH1* do not appear to impact genome-wide H3K36me2, but specifically reduce H3K36me2 levels at homeotic developmental genes, suggesting that the presence of additional factors can release the auto-inhibition on ASH1 [150].

- (II) Residues that recognize and stabilize the H3 N-terminus in the catalytic groove of the enzyme

Various structural analyses have helped to uncover the extensive interaction between the histone H3 tail peptides and the H3K36 methyltransferases. Here, we will mainly limit our discussion to Set2/SETD2. Consistent with the view that the AWS and SET domains are functionally sufficient for associating with the histone H3 tail, an extensive array of hydrogen bonding (H-bond)—both direct and water-mediated—and hydrophobic contacts are formed between residues of the AWS-SET-post-SET domains of SETD2 (also NSD2 and NSD3) and the H3K36M peptide [75, 119, 145, 146] (Fig. 4).

SETD2 (probably also Set2, as observed from similarities in the primary sequences) is endowed with differentially charged channels to accommodate the H3 tail, which is enriched with proximal, non-charged and distal basic and bulky residues relative to the Lys-36 residue [145] (Figs. 3, 4A and B). These interactions stabilize and position the K36 residue within a triangular core bounded by three juxtaposing arrays of *trans*-interacting β-sheets, constituting a pseudoknot-like structure that clamps the H3 tail (S31-K37) within the catalytic site, and positions the Lys-36 residue in proximity of the methyl donor (Figs. 1B, 4A) [145, 151].

Several conserved residues within SETD2 flank the histone tail in this catalytic space, especially Tyr-1579, Met-1607, Phe-1664 and Tyr-1666 with Met-36 of the peptide (where Met-36 is used in place of Lys-36) [145]. These residues potentially stabilize the interaction between the histone tail and the HMTase (Figs. 1B, 4B) by conferring proper positioning of the histone H3 tail and accommodating steric alignment of the K36 residue within the catalytic pocket. Similar residues were also found to embrace H3M36 in NSD2, except for a leucine residue (Leu-1120) in the place of methionine residue within the FMFY motif of SETD2 (FLFY in NSD2). Future mutagenesis studies would help to reveal whether this substitution—that differentiates between a di- and trimethylase—can have an impact on the formation of di- and trimethylation [109]. Upon complex formation, the Pro-38 of the H3 tail kinks out to accommodate Met-36 in *C. thermophilum* Set2 [75]. Pro-38 thus appears to act as a structural hinge to confer additional control over the catalytic pocket. In line with this view, Pro-38 was pliable to conformation change via *cis–trans* isomerization in budding yeast [152].

### Nucleosomal DNA unwrapping aids in H3K36me catalysis

NSD3 associates with the nucleosome with 12-fold higher affinity when it is wrapped with 187 bp of DNA over 147 bp of DNA, as shown through microscopic thermophoresis-based binding assays [119]. Through cryo-EM analysis, the 20-bp DNA linker termini peel away from the histone octamer surface at the dyad axis—where DNA enters and exits the nucleosome—permitting NSD3 to insert into the space between the unwrapped DNA and the core histones [119]. Similar unwrapping of DNA is also required for Set2 and NSD2 to bind H3K36 at the nucleosomal dyad axis [75, 109]. In studying NSD2, Sato et al. (2021) noted that H3K36 occupied a ‘congested’ environment co-occupied by two gyres of DNA, with unwrapping of the DNA displaces a gyre to facilitate access for NSD2 to H3K36 [109]. Thereafter, upon gaining access, the Arg-117 residue of Set2 stabilizes the displaced DNA via electrostatic interactions, which, in turn, result in rearrangement of the post-SET domain, probably to facilitate the conformation change required for the release of the auto-inhibitory L_IN_ loop [75, 145]. Similar stabilization of the DNA backbone was also observed with several lysine residues in NSD3 (Lys-1074, Lys-1077 and Lys-1080) to sustain full catalytic activity [119].

The series of enzyme–DNA interactions allosterically stack Lys-1234 of NSD3 (and Lys-1152 of NSD2) against histone H3Y41 (Fig. 4D), which is located at the junction between the H3 N-terminal tail and the histone fold domain [153, 154]; this is perhaps to restrict the mobility of the H3 tail for docking into the catalytic site of the enzymes in vivo. Future structural studies using longer H3 tail peptides will be required to verify this possibility within the nucleosomal context. Conversely, the enzyme–DNA binding, in turn, stabilizes enzyme–nucleosome binding, as observed for *C. thermophilum* Set2 [75, 109].

Taken together, these findings suggest that the binding site on the nucleosome at ground state is occluded by tight winding of DNA against the histone octamers. Upon binding with linker DNA, the HMTases displace the DNA gyres from the octamer surface, possibly in cooperation with chromatin remodelers such as Asf1, which can promote H3K36 trimethylation by Set2 [155]. The enzyme then wedges into the space that become accessible at the dyad axis. This binding between the enzyme and nucleosome then allosterically activates the enzyme by releasing the auto-inhibitory L_IN_ loop, permitting the insertion of H3 tail into the catalytic site in the formation of a stabilized enzyme–DNA-histone ternary complex.

### Roles of other histones in H3K36me catalysis

Using immunoblotting to check H3K36me levels in a collection of budding yeast strains hosting mutations in histone residues, Endo and colleagues pinpointed several residues within the histone H2A C-terminus (Gly-107, Ile-112 and Leu-117) and histone H3 αN helix (Thr-45, Arg-49 and Arg-52) that are critical for H3K36me. Subsequent computational modeling led the authors to propose the presence of a structured surface on the nucleosome constituted by these histones in the regulation of H3K36me3 [156]. This hypothesis is confirmed by the cryo-EM study that revealed a hydrophobic patch constituted by Ile-111, Leu-116 and Lys-119 in histone H2A carboxyl domain and Leu-48 and Ile-51 in the αN helix of histone H3, which form hydrophobic and electrostatic interactions with Asp-146, Ile-150, and Ala-153 in the AWS domain of *C. thermophilum* Set2 [75]. Mutations of these histone residues abolished H3K36me, which was not observed in mutants of residues (H2A Gln-114 and Asn-115) that do not interact with Set2 [157], supporting the functional essentiality of the docking of Set2 onto the hydrophobic patch for mediating the catalysis of H3K36 [119].

Histone H2AK119 and H2BK120, which are situated within the H2A-H3 surface, can be ubiquitinated to modulate the binding of the HMTases via the AWS motif [75, 109, 119]. Artificially tethering ubiquitin to H2BK120 stimulates Set2 activity, without affecting the binding of Set2 on the nucleosome; this suggests that ubiquitin may modulate the orientation of H3K36 within the Set2 catalytic site [75]. Intriguingly, ubiquitination of H2AK119 and H2BK120 inhibited NSD3 activity, unlike that of Set2 [119], lending support to the role of ubiquitination in differentially regulating the degree of methylation on H3K36.

Set2 was also reported to bind histone H4 at Lys-44 [80]. Mutating H4K44 not only interfered with the H4–Set2 interaction, but reduced H3K36me2/3, increased H3K36ac, and enhanced transcription from cryptic promoters in gene bodies. However, these phenotypes were not observed for all H4K44 mutants: whereas K44Q and K44E disrupted H3K36me2/3, H4K44R and H4K44A did not, pointing to a more complicated role of H4. In fact, cryo-EM failed to detect a direct H4–Set2 interaction, and histone H4 is situated furthest from the dyad axis at which the enzyme (NSD3) sits on the nucleosome [119]. However, H4K44 was observed to orientate histone H2A C-terminus within the hydrophobic surface, likely to affect Set2 activity [75].

In summary, H3K36 HMTase Set2 (and also NSD2 and NSD3) interacts with DNA and the histone H2A-H3 surface to release the auto-inhibition and extended region of the H3 tail to position H3K36 for catalysis, which may bear consequences that impact the number of methyl groups that can attach to H3K36.

## “Decision-making” motifs and residues in Set2 regulate the degree of methylation

Yeast Set2 catalyzes all forms of H3K36 methylation [12, 36, 158]. There have been multiple theories put forward to explain how Set2 regulates the degree of methylation (mono, di or tri). In fission yeast Set2, the SRI domain and a newly defined “domain of unknown function” (DUF) located proximal to the SRI domain [12] are two of the key motifs implicated in determining the H3K36me3 methylome. Indeed, deletion of the SRI and DUF domains from fission yeast Set2 causes a considerable reduction in total H3K36me3 levels without affecting H3K36me2 [12]. In contrast, loss of the SRI domain in budding yeast diminishes both H3K36me2 and H3K36me3 marks [79]. This discrepancy may be related to the speculative differences in binding abilities between budding yeast Set2 and fission yeast Set2 in their association with RNAPII [159]. An additional C-terminal auto-inhibitory domain located between the SRI and SET domains in both human and budding yeast Set2—but not in fission yeast—may also play a role in global methylation levels. This auto-inhibitory domain controls the DNA binding activity of the SRI domain and fine-tunes the involvement of Set2 in H3K36 methylation [78]: its deletion in budding yeast Set2 leads to a specific loss of H3K36me3, similar to that measured following deletion of the SRI domain in fission yeast Set2 [33].

H3K36me2 to H3K36me3 conversion depends on the SET domain, which encompasses the conserved residues Arg-1625 and Phe-1664 in human SETD2, Arg-315 and Phe-359 in fission yeast Set2, and Arg-195 and Phe-234 in budding yeast Set2. These residues position the H3K36me2-containing histone H3 tail [158]. The methyl group donor, S-adenosyl-L-methionine (SAM), then facilitates the transfer of an additional methyl group to the protein, resulting in trimethylation [57]. Through crystallography analysis, it was suggested that Phe-1664 of SETD2 lies near the histone H3 tail, lending further support to the importance of this residue in regulating the methylation status of H3K36 [160] (Fig. 4A). Consistently, mutation of Arg-1625 in SETD2 (R1625C) in ccRCC attenuates the level of H3K36me3 relative to that found in cells expressing the wild-type SETD2. However, the contribution of this R1625C mutation to the switch in H3K36 methylation is unclear, as the mutation causes a reduction in both SETD2 protein and mRNA levels and leads to a shortened SETD2 protein half-life [57]. The R1625C variant of SETD2 also has reduced substrate-binding capacity and H3K36 trimethylation catalytic activity, which may be linked to a disruption in the hydrogen bonding network arising from possible steric clashes near the SAM binding site [57].

Amino acid substitutions, such as Phe-to-Tyr or Tyr-to-Phe, within the catalytic domains of HMTases have an impact on histone substrate selectivity at different methylation states. Indeed, replacing Phe with Tyr results in steric clashing within the enzymatic site, thereby preventing the transfer of a third methyl group onto the dimethylated lysine residue. Conversely, a Tyr-to-Phe change creates more space, which can encourage trimethylation [161, 162]. A recent study took advantage of this principle, and mutated Phe-234 to Tyr in budding yeast Set2 (Phe-1664 in SETD2). The resultant Set2-F234Y mutant caused a selective reduction in H3K36me3 without significantly affecting the status of H3K36me1/2; conversely, a Tyr-to-Phe mutation at Tyr-149 within the SET domain of Set2 (Y149F) led to diminished H3K36me1/2 without affecting H3K36me3 levels [158].

Closer scrutiny of these conserved SET domains shows the presence of several Phe and Tyr residues, and raises the possibility that fine-contour modulation of the SET domain could influence the contact between catalytic pocket and the histone H3 tail and, in turn, affect the methylation status of H3K36. Several of these residues are only conserved in fission yeast, budding yeast, and fly (e.g., Phe-179, Phe-185, Phe-234, Tyr-244, fission yeast numbering) but not in SETD2 (Fig. 1A), which may suggest a structural basis for the enzymatic promiscuity of these Set2 homologues in catalyzing all three forms of H3K36me. Further structural and mutagenesis studies will be required to confirm these hypotheses.

As mentioned above, screening of the histone mutants that resulted in H3K36me disruption identified that residues of the hydrophobic patch on histone H2A (Gly-107, Ile-112 and Leu-117) and of the αN helix of histone H3 (Thr-45, Arg-49 and Arg-52) specifically disrupt H3K36me3 but not H3K36me2 or H3K36me [156]. It is therefore possible that Ile-150 and Ala-153 of *C. thermophilum* Set2 [75]—or equivalent residues in Set2/SETD2 from other species—may be involved in regulating the H3K36me2-to-me3 switch.

## Interplay between H3K36me and other chromatin modifications in transcriptional regulation

**Fig. 5:**
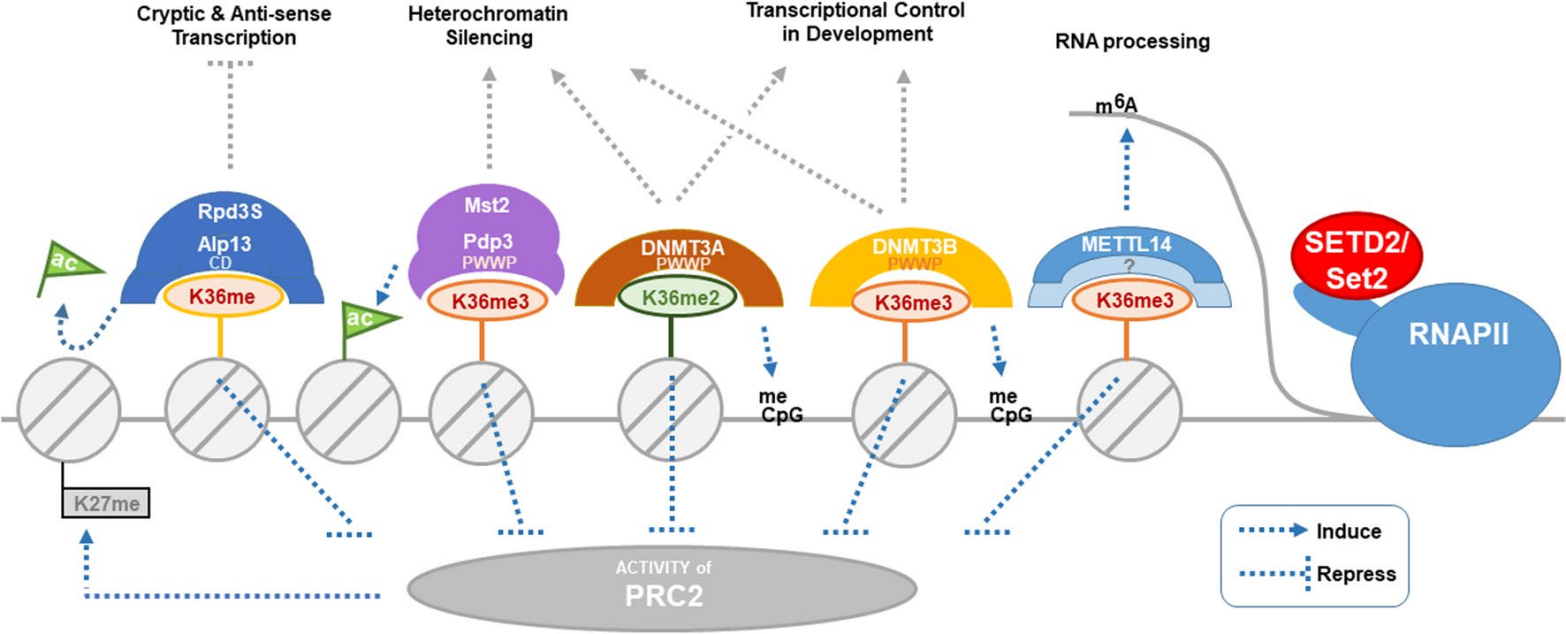
Proposed crosstalk between di- and tri-methylated H3K36 and other modifications on histones, DNA, and RNA. Abbreviations: K36me: di- and/or tri-methylated H3K36, K36me2: dimethylated H3K36, K36me3: tri-methylated H3K36, ac: histone acetylation, CD: chromodomain, PWWP: proline-tryptophan-tryptophan-proline motif, m^6^A: N^6^-methyladenosine RNA modification, RNAPII: RNA polymerase II, meCpG: methylated cytosine in CpG motif on DNA. Rpd3S-K36me interaction is observed in human, budding yeast and fission yeast; Mst2-Pdp3-K36me interaction is reported solely from fission yeast system thus far, while interactions of DNMTs and MTTL14-K36me are studies from human

Set2-mediated methylation of H3K36 depends on its physical interaction with RNAPII and its genetic interaction with other transcriptional elongation factors, including the histone chaperone, Spt6 [159]. Recent research has revealed interesting roles for the different types of H3K36me in terms of crosstalk with other chromatin PTMs, as well as with DNA and RNA templates. The interactions between H3K36me marks and the associated reader protein(s) determines the impact of H3K36me on transcription and other cellular functions. MRG15 is a multifunctional chromatin organizer that reads H3K36 marks by binding to H3K36me3 via its chromodomain to affect exon definition and splicing through the recruitment of downstream effectors (Fig. 5) [163]. Comparatively, DNA methyltransferase (DNMT) binds to H3K36me3 via its PWWP domain to control DNA methylation (Fig. 5). Eaf3, on the other hand, interacts with H3K36 via its Tudor, PWWP, and chromodomain to prevent aberrant transcription, and Phf1 and Phf19 in PRC2 interact with H3K36me3 via their Tudor domains to orchestrate H3K27 methylation (Fig. 5) [164, 165]. These interactions purportedly fine-tune the dynamic recruitment of transcription-related factors for different phases of transcription [166–168]. In the next section, we explore the recent insights into how H3K36me crosstalk with DNA methylation, histone PTMs (acetylation and H3K27 methylation), and N^6^-methyladenosine modifications on the RNA transcript.

## Antagonism between H3K36me and H3K27me

Trimethylated Lys-27 on histone H3 (H3K27me3) confers silencing, particularly on developmentally regulated genes. H3K27me3 and H3K36me2/3 are mutually exclusive on the same histone H3 and, consequently, do not co-occur across most of the genome [169–171]. Such H3K27me/H3K36me antagonism plays important role in setting up developmental programs [169, 172]. Indeed, disruption of H3K36 HMTase activities in worm, fly and *Neurospora crassa* (*N. crassa*) results in aberrant genome-wide H3K27me3 distribution, leading consequently to developmental defects that arise from transcriptional de-repression of genes that are typically repressed by downstream effectors of H3K27me3 silencing, such as Polycomb group proteins [173–175]. Studies performed in human glioma cells further point to the formation of a boundary by H3K36me2/3 to prohibit the spread of H3K27me3 on chromatin [176]. Consistent with these observations, myeloma cells overexpressing the H3K36 di-methylating MMSET HMTase exhibit hypersensitivity to an inhibitor of the Polycomb repressive complex 2 (PRC2) H3K27 HMTase [177].

In HeLa cells and in the human genome, when histone H3 is unmethylated on H3K36 it tends to be mostly methylated on H3K27; this is except for any newly synthesized histone H3. Furthermore, the enzymatic activity of PRC2 is inhibited on H3K36 pre-methylated nucleosomal substrates [171]. This inhibition requires the presence of H3K36me2/3 (or H3K4me3) in *cis* on the same or opposite histone tail hosting the H3K27 substrate [169, 171, 178]. H3K36me2/3 does not counteract docking of PRC2 onto histone H3 but, rather, inhibits its catalytic activity [170, 179]. Through mutagenesis analysis, a H3K36me-sensing motif—D^630^PVQK^634^—was uncovered on the catalytic EZH2 subunit of PRC2. This motif forms a solvent-accessible pocket for the insertion of an unmodified H3 tail [170]. Using cryo-EM, H3K36 was shown to be strategically situated at a critical site that permits the correct alignment of the histone H3 tail along the EZH2 interface to ensure H3K27 methylation [179].

The antagonism between H3K36 and H3K27 methylation is envisioned to stabilize epigenetic states in vivo, which is essential to target chromatin modifiers for enforcing transcriptional specificity in key developmental genes [172]. In line with this view, sequencing of cancer patient genomes revealed mutually exclusive occurrence of K27M and K36M mutations in histone H3.3 genes in several pediatric cancers, with K27M alteration found in brain tumors (pediatric diffuse pontine glioma and pediatric glioblastoma) [180, 181] and K36M mutation in bone tumors (chondroblastoma, chondrosarcoma and giant cell tumor of bone) [182, 183]. Loss of H3K36me in undifferentiated sarcoma cells is correlated with upregulation of H3K27me preferentially at intergenic regions. These ectopically induced H3K27me is proposed to compete for binding of reader proteins with H3K27me-enriched gene loci, leading to the transcriptional de-repression of these genes [183]. Various mutants of H3.3-Gly-34 were also identified in these pediatric cancers [180, 182]. Study of ectopically expressed G34L and G34W-mutated histone H3.3 proteins in HeLa cells revealed reduced H3K36me3 and increased H3K27me3 in *cis*, which correlated with augmentation of H3K27me3-interacting PRC1 and PRC2 complexes and obstruction of H3K36me3-reader ZMYND11 binding at the incorporation sites of these Gly-34-mutated histone H3.3. These observations show that H3K27me–H3K36me balance fine-tune localization of effector proteins to enforce epigenetic programs at specific genomic loci [172, 184].

Even though there is a general exclusivity of H3K27me and H3K36me on the genome, a small proportion of nucleosomes was, however, detected via mass spectrometry to contain both H3K36me2 and H3K27me1/2/3. Computational modeling supported the co-occurrence of these usually mutually exclusive marks in the same nucleosome if Lys-27 is first methylated on the histone H3 tail before Lys-36. This prediction is consistent with the reported evidence that H3K36me3 directly inhibits EZH2 methylation of H3K27, whereas similar evidence has not been reported for the reverse scenario [169–172, 178].

## Regulation of histone acetylation by H3K36me

H3K36me2 and H3K36me3 tend to show a higher enrichment within gene coding sequences [185–189] with H3K36me2 preferentially enriched closer to the transcriptional start site than H3 K36me3. The level of H3K36me2 appears to be less correlated with transcription than H3K36me3 [190]. Set2-mediated methylation of H3K36 acts at the end of the coding sequence in a negative feedback mechanism to slow RNAPII transcription; this action counteracts histone acetylation, thereby compacting chromatin to limit RNAPII accessibility, via the recruitment of the Rpd3S histone deacetylase (HDAC) complex [191]. The Rpd3S HDAC complex employs two of its subunits—Rco1 and Eaf3—to engage H3K36-methylated nucleosomes [192]. The recruitment of Rpd3S also depends on elongation factor Spt4/5 (yeast equivalence of human DSIF) and association with phosphorylated RNAPII CTD (Fig. 4) [193]. The targeting of HDAC complexes to localities of H3K36me counters histone acetylation, which promotes the formation of a less compacted chromatin conformation besides serving as the binding sites for recruiting transcriptional activators [194, 195]. Deacetylation of chromatin represses transcription of antisense and aberrant non-coding transcripts that arise from cryptic promoters in the wake of the elongating RNAPII, especially in genes with long coding sequences [196, 197, 198].

Eaf3–H3K36me interaction is essential for keeping the Rpd3S complex active (Fig. 5) [193]. Eaf3 binds H3K36me at a shallow histone binding surface cleft comprising a C-terminal α-helix and a β-barrel core, in which the conserved Tyr and Trp residues (Tyr-23, Tyr-81, Trp-84 and Trp-88 for budding yeast Eaf3) confer selective recognition of H3K36me3 [192]. This selectivity is imposed by a conformational switch that occurs when the Rpd3S complex contacts chromatin: this contact leads to allosteric enhancement of the binding strength of the CD on H3K36me2/3 and is thought to constitute one of the strongest chromatin–protein interactions documented to date [32, 35, 192, 199]. H3K36me3 and the yeast Eaf3 homolog , Alp13, also co-localize on heterochromatin in the fission yeast genome during S-phase, when the heterochromatic repeat sequences are preferentially transcribing [200, 201]. Deletion of *alp13* or *set2* in budding and fission yeast increases antisense transcription in euchromatin and heterochromatin regions of the genome [33, 200, 202–204], indicating a conserved interaction among Eaf3/Alp13, Set2, and H3K36me across different species; albeit, a direct interaction between Alp13 and H3K36me is yet to be structurally determined.

The transcription spike is accompanied by the momentary de-repression of the non-coding repeats at constitutive heterochromatic loci induces the activation of RNA interference during S-phase of the cell cycle, along with high levels of histone acetylation [200]. These events suggest the possibility that HAT, including the Mst2 complex, may be targeted to heterochromatin in normal cell cycling. H3K36me, which functions to coordinate non-coding sequence transcription during S-phase, will temporarily co-localize with the HAT and HDAC effectors, which possess opposing enzymatic activities on histone acetylation [200, 205]. It is possible that H3K36me can maintain a dynamic equilibrium wherein transcriptional activation is fine-tuned in conjunction with chromatin packaging to accommodate the needs of chromatin unraveling during DNA replication and transcription [154, 200]. During S-phase, although heterochromatin becomes less compacted, it is rarely completely disrupted (as in mutants of heterochromatin factors, such as that of H3K9 HMTase). H3K36me may facilitate the maintenance of the partially opened state of heterochromatin during the DNA replicative phase by regulating a balance between silencing and anti-silencing activities in conjunction with other chromatin factors and histone modifications. For instance, crosstalk is likely to be established with H3Y41 phosphorylation (H3Y41p), which functions to differentially modulate chromatin binding of CD-containing proteins. The release of Swi6/HP1 and the recruitment of RITS component Chp1—both CD-containing proteins—by H3Y41p extends the duration in which centromeric heterochromatin becomes accessible to RNAPII to transcribe underlying non-coding repetitive sequences [154, 206].

Apart from recruiting the Rpd3S HDAC complex, H3K36me3 also anchors the Mst2 H3K14 histone acetyltransferase (HAT) complex to euchromatin to prevent HAT from being mistargeted (Fig. 5); mistargeted HAT purportedly antagonizes silenced chromatin at the heterochromatic loci in fission yeast [201]. The budding yeast Sas3-dependent NuA3 complex and fission yeast Mst2 complex (equivalent to human MYST3 complex) is localized to H3K36me3 via its PWWP domain within the Pdp3 subunit and Yng1 subunit, respectively [207, 208]. A mutation at residue Phe-109 of the PWWP domain (F109A) abolishes the interaction between the Mst2 complex and H3K36me3; this allows HAT to be misplaced from the euchromatic gene loci and leads to aberrant activation and transcription of heterochromatic sequences, along with a dissolution of transcriptional silencing [207].

## Interactions between H3K36me and DNA methylation

DNA methylation is important for heterochromatin formation not only in mammals, but also in some yeast species [209, 210]; consistently, DNA methylation occurs on 0.3%-1% and 3%-8% of total cytosine (C) residues in yeast and mammalian (of total C) genomes, respectively [211, 212]. DNA methyltransferase 3A (DNMT3A) and DNMT3B in mammals and Pmt1 in fission yeast are capable of catalyzing methylation at the 5’-position of cytosine (C) residues on the CpG nucleotide pair [212, 213].

A specific interaction occurs between H3K36me2 and the N-terminus of the PWWP domain of DNMT3A (Fig. 5): DNMT3A is recruited onto nucleosomal or linker DNA where its enzymatic activity is stimulated [40, 210, 214]. Studies show that mutation of the conserved Lys-295 residue on the DNA-interacting surface of the PWWP domain in DMNT3A disrupts its binding to both DNA and H3K36me2, leading to an aberrant sub-nuclear localization of the DNA methyltransferase [210]. Yet, unlike the recognition of H3K36me2 by DNMT3A, the PWWP domain of DNMT3B preferentially binds to SETD2-induced H3K36me3 to anchor itself onto gene bodies in CpG-rich regions [210, 215]. Indeed, a genome-wide increase in H3K36me2 levels correlates with an increase in nucleosomal 5-methylcytosine levels; this, in turn, is correlated with an overexpression of the NSD2 (Nuclear Receptor Binding SET Domain Protein 2) cancer-driver and an upregulation in genes enriched in oncogenic pathways [24]. However, removal of genomic H3K36me2 via the concurrent knocking down of NSD1 and NSD2 results in a genome-wide reduction in DNA methylation at intergenic loci, along with hypersensitivity to the DNA-hypomethylating agent decitabine. In contrast, *Setd2-*knockdown cells, which become depleted of H3K36me3, show no decitabine sensitivity, providing evidence for the differential regulation of H3K36me2 and H3K36me3 on DNA methylation [216].

## Interaction of H3K36me with RNA N^6^-methyladenosine (m^6^A) modification

N^6^-Methyladenosine (m^6^A) is an abundant modification imposed on messenger RNA (mRNA), ribosomal RNA (rRNA), and small nuclear RNA (snRNA) of higher eukaryotes [217, 218]. A stable and accurate level of m^6^A modification ensures proper nuclear RNA export and splicing, mRNA stability, circular RNA translation, microRNA biogenesis, and long non-coding RNA metabolism. This type of methylation is also physiologically implicated in counteracting obesity, immune dysregulation, the generation of meiotic defects and carcinogenesis [219–221].

The METTL14 methyltransferase, responsible for m^6^A modifications, directly binds H3K36me3 (Fig. 5) and methylates adjacent nascent RNAPII transcripts; in the human transcriptome, this was observed at more than 12,000 loci of consensus RRACH sequences ([G/A][G > A]m6AC[U > A > C]) [222–225]. Most m^6^A peaks are close to stop codons or 3’ untranslated regions, with the methylation status fluctuating along with meiosis events and cellular stress [226]. m^6^A marks on mRNA transcripts can be significantly depleted by SETD2 knockout, R1625C mutation in the SET domain, or a disruption in the RNAPII-interacting SRI domain of SETD2 [227]. It remains unknown how METTL14 recognizes H3K36me3 for specific mRNA methylation, as no known H3K36me3-interacting domains have been identified from the METTL3–METTL14 complex crystal [228]. Yeast Set2 is also believed to interact with a defined group of nascent RNAPII transcripts, and this interaction likely affects chromatin occupancy in a manner similar to the related protein, Set1. It has been hypothesized that RNA binding structurally stabilizes Set2 chromatin binding [229].

## Conclusions and future questions

The structural and physiological studies reviewed here show that HMTases, including NSD2, NSD3, Set2 and SETD2, possess structural features across multiple domains that confer the catalytic specificity of di- and tri-methylated histone H3K36. These HMTases act in conjunction with various nucleosomal determinants and via crosstalk with other modifications and transacting factors. The selectively of H3K36me2/3 elicits specific physiological outcomes in the chromatin context in conjunction with co-occurring modifications on histones, DNA, and RNA, as well as various transacting factors. However, many questions remain to be answered. For example: (1) What is the sequence of events concerning the interactions of the enzymes with DNA and histone H2A/H3 hydrophobic patches in terms of stimulating the release of the auto-inhibitory loop, and how the latter can further potentiate stronger binding? (2) How is (1) reversed after catalysis is completed? (3) How does the ubiquitination of histone H2A and H2B contribute to the selectivity of di-to-trimethylation? (4) What are the critical residues within the full-length enzyme(s) that influence di-to-trimethylation within the catalytic AWS-SET-post-SET domains and beyond (such as DUF, AID and SRI)? (5) What is the impact of these structural mechanisms on various H3K36me-regulated cellular processes? And, finally, (6) Does the disruption of any of these regulatory pathways underpin the cancer-driving capability of the mutated HMTases in various cancers? Future structural studies and in vivo analyses are expected to provide the answers to these questions.

## Data Availability

Data are depicted in the submitted figures. No material was involved in this work.
